# Living with wolves: A worldwide systematic review of attitudes

**DOI:** 10.1007/s13280-024-02036-1

**Published:** 2024-06-04

**Authors:** Magnus Barmoen, Kim Magnus Bærum, Kristin E. Mathiesen

**Affiliations:** 1 Faculty of Applied Ecology, Agricultural Sciences and Biotechnology, Inland Norway University of Applied Sciences, Campus Evenstad, 2480 Koppang, Norway; 2 Norwegian Institute for Nature Research, Fakkelgården, 2426 Lillehammer, Norway

**Keywords:** Human dimensions, Interaction modelling, Large carnivores, Tolerance, Wildlife conflict, Wolf

## Abstract

This systematic review of peer reviewed articles on attitudes towards gray wolves (*Canis lupus*), shows that attitudes are mainly measured either by *mean values* of attitudes or by *proportional differences* in attitudes. This may impact on how attitudes are perceived and interpreted across studies and areas. However, independent of method used, we found that people living in areas where wolves always have existed, are more negative towards wolves compared to people living in areas where there are no wolves, or where wolves have recovered after years of absence. People who express fear, or being directly affected by having wolves, such as farmers and hunters, report more negative attitudes compared to other groups of respondents. For wolf conservation we recommend politicians and management authorities to prepare local societies of the different consequences of living in wolf areas. We recommend using dialogues and conflict management methods to minimize the level of conflicts.

**Supplementary Information:**

The online version contains supplementary material available at 10.1007/s13280-024-02036-1.

## Introduction

Large carnivores and humans have a long history of being at odds with each other (Fritts et al. 2003), as the presence of large carnivores may entail conflicts of interest in resources and land use with people who live in the same areas (Kansky et al. 2014; van Eeden et al. 2018; Lozano et al. 2019). Some species, such as gray wolves (*Canis lupus*), are overrepresented in such conflicts. Wolves are often defined as a flagship species which acts as a symbol that can increase attention and contribute to the conservation of the system (ecosystem) in which the species represent or live in (Heywood and Watson 1995). Additional of being part of social conflicts (Nie 1999; Douglas and Veríssimo 2013; Slagle et al. 2019), wolves are also described as iconic (Kellert et al. 1996; Lynn 2010), or linked to stories and old myths (Johnson 1974; Varga 2009; Jürgens and Hackett 2017). Wolf representations, however, depend on the eye of the beholder, and social representations, such as whether or not the species belongs in an area, may vary greatly even between people living in the same areas (Figari and Skogen 2011; Peterson et al. 2020). Accordingly, a range of emotional responses towards wolves are observed today, from blind love to raging hate, leading to diverging opinions on management goals and actions (Wilson 1997; Slagle et al. 2019). Unsurprisingly, wolf-human interactions have been a focus in the scientific literature for decades, where researchers aim to better understand the roots of the conflicts, and why people perceive wolves in the way they do. However, while single attitude surveys in restricted geographical locations can contribute knowledge on attitudes towards wolves in that particular time and space, they fall short if we are interested in more general trends. The meta-analysis conducted by Dressel et al. (2015), compared attitudes towards bears (*Ursus arctos*) and wolves across Europe. They found more positive attitudes towards bears than wolves, and that attitudes towards wolves even got less positive the longer people and wolves co-existed (Dressel et al. 2015). In this study, we expand the study done by Dressel et al. (2015) to not be restricted to Europe but have utilized the accumulated literature worldwide in the period 1980–2020.

From being one of the most widely distributed non-human land mammals worldwide (Young and Goldman 1944), wolves have been extirpated from many countries due to human persecution and habitat fragmentation (Ripple et al. 2014; Hunter 2019). Wolves killing livestock, as well as causing fear of attacks on humans, motivated the extermination (Mech and Boitani 2003). The development of protection and effective policy implementation in recent decades, has allowed large carnivores to recover in both Europe (Linnell et al. 2001; Trouwborst 2010; Kaczensky et al. 2013; Chapron et al. 2014) and North America (Linnell et al. 2001; Wydeven et al. 2009; Mech 2017). In the 1970s, environmental movements provided the motivation for pan-European legislative agreements, including the 1979 Bern Convention administered by the Council of Europe (Council of Europe 1979; Trouwborst 2010). In 1992, wolves became legally protected as part of the Habitat Directive, which covers all European Union member states (Council Directive (EEC) 1992). The EU member states counted 12 states at that time, and included Belgium, France, Germany, Italy, Luxembourg, Netherlands, Denmark, Ireland, UK, Greece, Spain and Portugal. The agreement of the Habitat Directive facilitated wolves to recover in parts of France, Germany, Switzerland, Denmark and Scandinavia as individuals migrated from large populations in Spain, Italy, Russia and Eastern Europe (Mech and Boitani 2003; Chapron et al. 2014). In North America, wolves were exterminated from all the contiguous U.S. states except Minnesota and Michigan (Young and Goldman 1944; Mech and Boitani 2003). However, the Endangered Species Act of 1973, and consequent protection and management by the US Fish and Wildlife Service have facilitated an increase in recovery in several states (Smith and Bangs 2009). This protection, in addition to dispersal has led to wolves are now occurring in states where they were previously considered locally extinct (Treves et al. 2009; Jimenez et al. 2017). Wolf distribution and abundance are less well known in Asia (Honghai 1999). In China, wolves are present in all provinces on the mainland, however large populations only remain in northwestern parts of the country (Mech and Boitani 2003; Wang et al. 2016). The number of reports of injury and loss caused by wolves to livestock has increased in recent years, followed by an increase in conflicts associated with the species (Zhang et al. 2010; Li et al. 2013). In northern Inner Mongolia, the distribution and abundance of wolves have been greatly reduced because of human interests in keeping predation of livestock and gazelles under control, yet few places have experienced complete eradication (Maruyama et al. 1996; Mech and Boitani 2003). In Russia, wolves were never eradicated, mainly due to their vast range, although variations in abundance and distribution occur caused by human influence and variations in wildlife prey abundance (Bibikov 1994; Fritts et al. 1994; Honghai 1999). After the collapse of the Soviet Union, wolves experienced a rapid increase in numbers, most likely because of the ending of governmental population control (Bragina et al. 2015). In summary, wolves are reappearing in former areas all over the world, but to a lesser extent in Asia where extensive changes in legislation have yet to take place.

As wolves recover in human-dominated landscapes, personal experiences of interacting with them increase, and conservation demands land sharing between wolves and humans (Treves and Karanth 2003; Linnell 2015; Cretois et al. 2021). However, wolves are still not present everywhere, so the costs of living with them are unevenly distributed (Karlsson and Sjöström 2007; Eriksson et al. 2015). While wolf recovery is being welcomed by people who perceive it as a contribution to the wilderness (Sjölander-Lindqvist 2008, 2009; Slagle et al. 2019), it is opposed by those who perceive wolves as a potential threat to livestock, big game populations and the rural way of life (Wilson 1997; Eriksson 2016; Mykrä et al. 2017). Successful management of large carnivore must balance different priorities and expectations among people of different beliefs and take into account changes and diversifications of stakeholder values (Dietsch et al. 2016; Bruskotter et al. 2017).

Attitudes can be defined as “a disposition or tendency to respond with some degree of favorableness or not, to a psychological object” (Fishbein and Ajzen 2010). Attitude towards an object determines a person’s willingness to behave in a certain manner (Manfredo 2008; Vaske and Manfredo 2012). Several scholars have divided attitudes into two main components, cognitive and the affective components (Eagly and Chaiken 1993; Fishbein and Ajzen 1977; Verplanken et al. 1998). Knowledge about public attitudes towards large carnivores such as wolves can help to predict the social foundation for future conservation (Bright and Manfredo 1996; Bruskotter et al. 2009; Vaske and Manfredo 2012), and determine whether carnivore–human interactions are expressed as conflict or coexistence. Coexistence has several definitions (Carter and Linnell 2016; Chapron and López-Bao 2016; Morehouse and Boyce 2017), but will in general reflect a tolerant attitude (Frank 2016). Positive attitudes associate with the valuing and respect of wildlife and a will to forego one’s own interests to benefit wildlife. Neutral responses could reflect lack of interest or no willingness to take action in response to a wildlife issue (Frank 2016), while negative attitudes may cause negative impacts on wildlife conservation by for example an increased acceptance of poaching (Liberg et al. 2012; Gangaas et al. 2013). Negative attitudes can also induce public resistance to conservation plans and policies and promote eradication policies (Zinn et al. 1998; Bruskotter and Fulton 2012).

Attitudes are generally fairly stable but can change rapidly (Eagly and Chaiken 1993; Heberlein 2012) when the benefit of change exceeds the costs of keeping that attitude. Direct experience can cause increased attitude strength, as well as changes in them (Fazio et al. 1983; Petty et al. 1997). Previous studies have shown negative attitudes to be associated with both direct experience (i.e. loss of livestock or damage to property, observations of wolves or tracks; Eriksson et al. 2015) and indirect experiences (i.e. experienced by others; Karlsson and Sjöström 2007). In the general public, attitudes towards wolves are likely to be held loosely as they are based largely on second-hand information and no direct experiences (Ericsson and Heberlein 2008). Consequently, these attitudes could be subject to change when people experience the return of wolves to their area (Ericsson and Heberlein 2003; Ericsson et al. 2008; Treves et al. 2013). Based on their different experiences with wolves, some social groups tend to be associated with negative attitudes towards wolves, e.g. farmers (Kellert 1985; Bright and Manfredo 1996; Kleiven et al. 2004) and hunters (Ericsson and Heberlein 2003; Mykrä et al. 2017).

Our main objective is to test whether attitudes towards wolves are influenced by the presence of wolves and how this may vary between different respondent groups included in the surveys. We want to determine whether people hold more negative attitudes in areas in which wolves have been continuously present compared to those where wolves have returned after being locally extinct.

## Materials and methods

### Search protocol and process

We conducted a systematic review of English language scientific peer-reviewed journal articles measuring attitudes towards wolves published during the 40-year period between 1 January 1980 and 31 December 2020. From our initial search, 1980 seemed to be a natural starting point as few peer-reviewed studies were available before then. We searched for articles using two electronic databases: Web of Knowledge and Scopus, and followed the guidelines for systematic reviews provided by Pullin and Stewart (2006). We considered publication bias (i.e. strong bias to publish studies that show significant results) to be unlikely within our literature search, as performing significance-tests for attitudes towards carnivores seems counterproductive. As such, we did not carry out any formal assessments of possible publication bias. We used the following commonly used search terms: “attitude* OR tolerance* OR perception* OR judgement* AND wolf* OR wolves* OR canis lupus”. We restricted the inclusion criteria to include quantitative studies on self-reported attitudes towards wolves only, to allow comparisons across studies. We only included studies that measured attitudes towards wolves, not surveys that exclusively measured support for specific conservation strategies, such as lethal control (e.g., Manfredo et al. 2020).

**Fig. 1:**
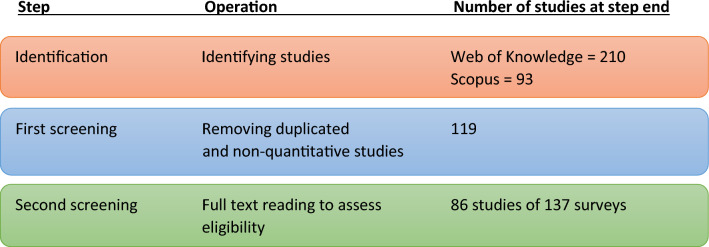
Steps in the methodological process to detect and include surveys that met the inclusion criteria

In the first step of the methodological process (Fig. 1), we identified 303 references that fitted the search terms. Through the first screening of articles, we excluded those that did not fit the inclusion criteria by briefly reviewing titles and abstracts, leaving 119 studies. In the second screening, we read the articles full text to assess their eligibility and commonly analyzed factors and research designs, as well as to establish categorization criteria. After removing studies that did not use quantitative examination of attitudes, applied previously published studies in their analysis, or did not report self-reported attitudes (e.g., analyzed media cover), the final dataset consisted of 86 studies (Appendix A). As a number of these articles (30) included sub-samples in which the authors either had conducted separate surveys in different locations or had reported data and information on the samples of different response groups, these were treated as separate surveys. Hereafter we use “study” for the number articles (n = 86) and “survey” for the separate surveys (n = 137). We based our analyses and summaries on the surveys.

### Data and data analysis

For the descriptive summary of surveys included in the review, the scale of the study area within each survey was categorized as local (municipalities and villages; see Alexander et al. 2015; Kirilyuk and Ke 2020), regional (counties or provinces; see Milheiras and Hodge 2011; Browne-Nunez et al. 2015), or national (includes studies in multiple countries; see Berg and Solevid 2015; Kaltenborn and Brainerd 2016). For descriptive purposes we chose to show surveys in four geographical regions: North America, Europe, Scandinavia (Denmark, Norway, and Sweden), and Asia. Scandinavia was shown as a separate entity from the rest of Europe because countries in this region were highly represented. We recorded general information about the respondents included in the surveys when available. This information varied among surveys but could for example include age, gender, income, education level, being a livestock owner, hunter and more (see the results section for the full list of relevant variables). Further, we recorded whether the variables tested were found to be non-significant, positively significant or negatively significant based on *p* values or showed negative or positive trends based on AIC values in the most parsimonious models. As many different variables were used, we chose to only report those that were included in at least five different surveys.

As the included surveys used different questionnaire designs, a variety of questions were used to measure attitudes towards wolves. Generally, the wording of the questions was fairly similar, such as “What are your feelings towards wolves?”, “How much do you care about wolves?”, and “Do you support conservation of wolves in your area?”. During the first screening, we noticed that surveys measured slightly different attitude objects. For example, “attitude towards wolves” and “attitudes towards wolf management”, as well as “attitude towards wolf management” and “attitude towards wolf restoration”. We tested for differences between the attitude objects. However, no significant differences between the attitude objects were found, and thus we treated all these possible attitude objects as one in the analyses.

The respondent groups were classified into groups similar to those used by Dressel et al. (2015): general public, students, urban public, public in wolf area, hunters, farmers (Appendix B). For analysis purposes, the urban public group were included in the general public group due to a low sample size. For the group students, we included researchers and managers both due to low sample sizes for the specific groups, and also with the rationale that these groups might usually be considered as influencing each other and share common attitudes in an academic environment. We thus renamed this new variable as Academics. The variable presence status was also based on Dressel et al. (2015), and described whether wolves were “Absent”, had “Returned” after being locally extinct, or had been continuously present “Persisting”. We simplified the variable compared to the one in Dressel et al. (2015) and merged the two categories “Far away” and “Close” into “Absent”, as both categories indicated that wolves were not present in the focal study area. We also merged two categories that described recolonizing populations, “Newly arrived” and “Established” into “Returned”. In addition, we added the category “Mixed” as some surveys included participants from more than one area that had different wolf presence status but did not report results separately for each status. Consequently, we used the following four categories: “Absent”, “Returned”, “Persisting, and “Mixed” (Table 1). In addition, as the sampling method varied across surveys, and could influence how results can be interpreted, we chose to differentiate between different sampling methods. Specifically, we used three categories: “Random sampling”, where any member of the population within the scope of the research frame may be selected; “Purposive sampling”, where the researcher selects a sample that is most useful for answering the research question of the survey; and “Convenience sampling”, where individuals who happen to be most accessible to the researcher are included. We also included snowball sampling, within the latter category, where participants are recruited via other participants.

**Table 1:** Here is an overview of the number of studies included in the review divided into the different response variables *Respondent groups*, *Presence status* (of wolves), and *Sample method*, and how the studies were distributed by geographical regions. The *Respondent groups* shows how many studies that were represented in the six different categories; Farmers, General public, Hunters, Local public, Public in wolf areas, to Academics. The *Presence status* shows how many studies that were done in areas were wolves were Persisting, Mixed (the study has mixed respondents living in areas with wolves being both present and absent), Absent or Returned. The *sample method* shows the distribution of the numbers of studies using different sampling methods represented in the different geographical regions

	Variable categories	Asia	Europe	North America	Scandinavia
Respondent groups	Farmers	12	5	7	1
	General public	0	7	18	11
	Hunters	0	4	10	3
	Local public	0	4	13	2
	Public in wolf area	8	4	6	7
	Academics	1	9	2	3
Presence status	Persisting	20	18	5	0
	Mixed	0	3	3	0
	Absent	1	3	23	0
	Returned	0	9	25	27
Sample method	Convenient	9	10	8	1
	Random	6	14	39	17
	Purposive	6	9	9	9

The way attitudes were measured varied between the surveys and included those using both single- and multiple-item indicators. Attitude measurements were provided in terms of favor or disfavor, like or dislike, and were measured on rating scales from three to nine points. Attitude scores were either reported as a proportion of respondents (hereafter proportional dataset) or as mean scores (hereafter mean dataset). Because of challenges with standardizing these two different measurements, we chose to analyze them separately. For proportional data (n = 56), Likert-scale measures were condensed into “negative”, “neutral”, and “positive” scores to allow for comparisons across surveys with different numbers of points. We used the percentage of respondents who expressed positive attitudes towards wolves as the dependent variable for these surveys. For mean data (n = 54), attitudes were reported as the respondents’ mean attitude score. The remaining 27 surveys reported attitudes both as a proportion and a mean. We chose to include those in the mean dataset, as this dataset had the lowest sample size. Consequently, the proportional data and the mean data included 56 and 81 surveys, respectively.

By using the attitude measurements in the proportional dataset, we created two separate variables. The first was named “positive proportion” and were created by scaling the positive attitude measurement scores into a numeric variable bound between 0 and 1, where 0 equated to no respondents being positive and 1 where all respondents were positive towards wolves. The second variable was named “negative proportion” and were created by scaling the negative attitude measurements into a numeric variable bound between 0 and 1, where 0 equated to no respondents being negative and 1 where all respondents were negative towards wolves. To test whether attitudes were associated with different wolf presence statuses and/or respondent groups, we fitted two models using the proportional data, one with “positive proportion” and one with “negative proportion” as the response variable. The mean data were scaled so that the value shared a common range between 1 (most negative) to 5 (most positive), across all surveys.

For the formal exploration of overall effects on attitudes, we choose to only include the two variables most relevant to the aims of this review, “presence status” and “respondent group”. This was due to the low sample size of surveys within the two measurements of attitudes, which restricted the number of variables to include in the statistical model. However, as there were multiple sampling methods used across the surveys, and the surveys could be considered as nested within these, we also included “sampling method” as a random effect in the model structure. Mixed-effects beta regression models with logit-links were constructed to explore the effects of the focal variables within the “proportional” data, using the glmmTMB-library (Brooks et al. 2017) in the statistical environment R (R Core Team 2020). For the “mean” data, linear mixed effect models were constructed using the lme4-library (Bates et al. 2015).

## Results

### Descriptive summary

**Fig. 2:**
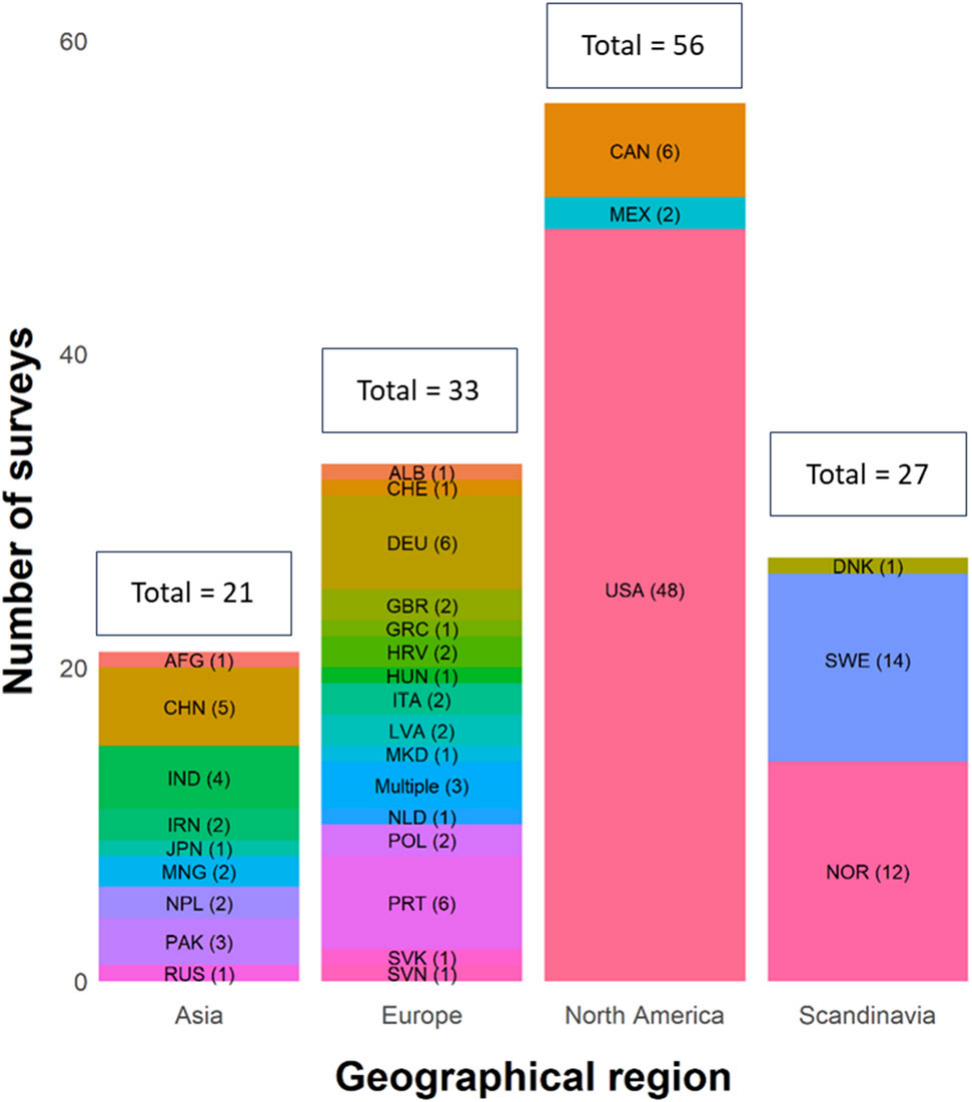
Number of studies included, by region and country. *AFG* Afghanistan, *ALB* Albania, *CAN* Canada, *CHE* Switzerland, *CHN* China, *DNK* Denmark, *DEU* Germany, *GBR* the United Kingdom, *GRC* Greece, *HUN* Hungary, *HRV* Croatia, *IND* India, *IRN* Iran, *ITA* Italy, *JPN* Japan, *LVA* Latvia, *MEX* Mexico, *MKD* North Macedonia, *MNG* Mongolia, *Multiple* study area comprise multiple countries, *NLD* the Netherlands, *NOR* Norway, *NPL* Nepal, *PAK* Pakistan, *POL* Poland, *PRT* Portugal, *RUS* Russia, *SVK* Slovakia, *SVN* Slovenia, *SWE* Sweden, *USA* United States of America

The 137 surveys included in this review represented all geographical areas in which wolves are present: North America (n = 55), Europe (n = 60), and Asia (n = 22) (Fig. 2). Out of the 60 surveys in Europe, 27 were conducted within Scandinavia. The three most surveyed countries were the United States of America (n = 48), Sweden (n = 14), and Norway (n = 12). In Europe, three surveys were conducted across multiple countries (Fig. 2).

**Fig. 3:**
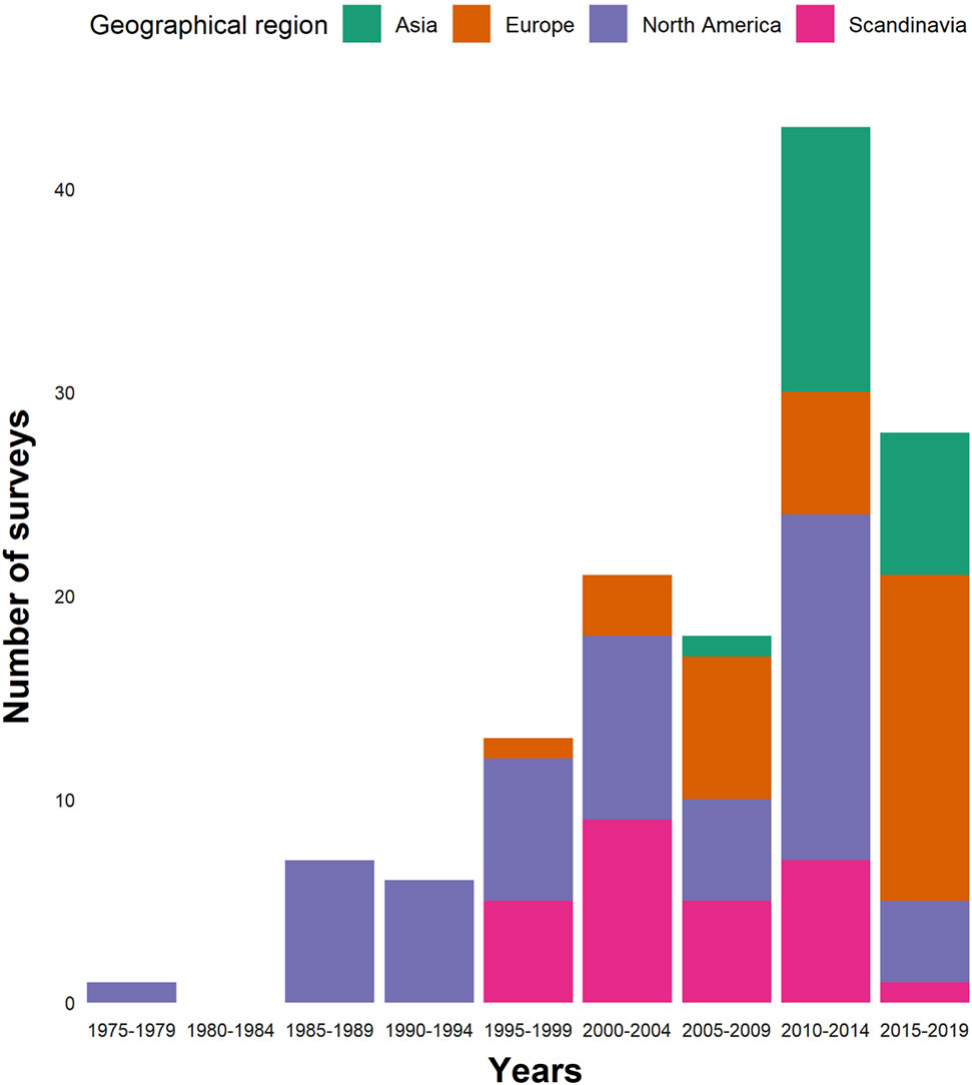
Number of studies per year of data collecting 1975–2019, given for 5-year periods. Colors depict the geographical region in which the surveys were carried out

One survey collected data before 1980 (Kellert 1985), yet was included as the year constraint in the inclusion criteria was based on publication year. Only eight surveys were carried out before year 1990, while 72 surveys were included from the last 10 years (2010–2019) (53% of total; Fig. 3). No surveys published within the study period collected data the year of 2020. The sample size of the surveys varied between geographical regions and ranged between 17 and 3142 respondents. Most surveys (93 out of 137), including all surveys from Asia, involved fewer than 500 respondents. However, 21 out of 137 had between 500 and 1000 respondents and 15 surveys had between 1000 and 2000 respondents. Only eight surveys involved more than 2000 respondents, of which one was from Europe (outside Scandinavia), two from North America and the rest from Scandinavia. In North America, most surveys were restricted to a single state (the United States of America) or province (Canada). Similarly, in Europe, most surveys were carried out at a regional scale, yet some focused on a local scale, restricting the scope to specific municipalities or villages. In Asia, several surveys were on a regional scale, but the majority were focused on local scales by restricting the scope to people living within one or a few closely located villages.

The way attitudes were framed and measured varied across the surveys. In 49 surveys (36%), we found reference to other surveys, such that the method used to measure attitudes was either copied or based on methods from other surveys (e.g., Kellert 1985; Kaczensky et al. 2004). While the oldest surveys, except Kellert (1985), measured attitude towards wolves, all other surveys in North America between 1980 and 2000 measured attitudes towards the restoration of wolves (Bath 1989; Tucker and Pletscher 1989; Kellert 1991; Lohr et al. 1996; Pate et al. 1996; Schoenecker and Shaw 1997). Outside North America, the oldest surveys from Scandinavia focused on attitudes towards the return of wolves among different respondent groups (Bjerke et al. 1998a, b; Kaltenborn et al. 1999). The oldest survey from Asia was published in 2010 and compared the compensation rules and practices between Solapur in India and Wisconsin in the US (Agarwala et al. 2010).

The most frequently used sampling method was random sampling (n = 76; 56%), while purposive sampling (n = 33; 24%) and convenient sampling (n = 28; 20%) were less used. The use of different sampling methods varied across geographical regions, with random sampling being the most common method for all regions except Asia (Table 1). For both Scandinavia and North America, random sampling was the most common by far, while for Asia all methods were almost as frequently used, although convenient sampling was used slightly more often. Overall, only 58 of the total 137 (42%) surveys reported response rate (number of respondents who answered the survey divided by the number of people assigned to the survey), which averaged of 61% and varied between 3.3 and 100%.

#### Summary of respondents in the surveys

Only 44 of the 137 surveys in total, reported the age of the respondents in some form. Of the 44, only 34 were comparable, as the remaining ten surveys reported age by variously defined categories or the proportion of respondents above a certain cut-off age, which were unique for each study. Gender was reported in 53 surveys, ranging from 30 to 100% male compared to female respondents. Education level was reported in 29 surveys but was reported in a variety of different ways making comparisons difficult.

#### Summary of variables influencing attitudes towards wolves

Education level, age and gender were the most frequently used variables to test for an association with attitudes towards wolves (Table 2). Age was found to be non-significant in most cases, although negative correlations were also commonly observed with older respondents holding more negative attitudes. For the twelve surveys testing for an influence of age on attitudes at the local scale, only one found a significantly negative effect. The remaining eleven found no effect. By contrast, at the regional scale, we found 19 surveys testing for the influence of age, where 50% showed a negative effect of age on attitudes and 50% showing no effect. For surveys done on large scale, the influence of age on attitudes was tested in eight surveys. We found a negative association in five of the eight studies, one with no effect, and two showed a positive association. For gender effects, the most common result was no observed trend. For those observing a trend, males were more often the most negative gender, yet the opposite were also observed. For education, two third of the surveys reported that higher levels of education correlated with more positive attitudes, while one third showed no association. Being a hunter resulted in all attitudinal outcomes, yet a significant correlation with negative attitudes was the most common. All outcomes were also observed among livestock owners, although a negative effect was most common. For fear and wolf area (i.e. respondent having wolf in residential area or not), only negatively significant or non-significant effects where found, with a significant negative effect being dominant for both variables. The variable testing for the effect of rural vs urban areas found both negative trend and no trend, while all outcomes were observed for the variable population size. The variable income showed all outcomes, too. While all outcomes were observed for the knowledge as well, positive trends and non-significant trends were most common (Table 2).

**Table 2:** Variables tested for their influence on attitudes in the reviewed surveys. Only variables included in at least five surveys are present. Column “not studied” gives the numbers of surveys that did not include the specific variable. “Positive”, “Negative”, and “No trend” give the number of surveys that found positive, negative and no effect for that variable, respectively. All rows sum to total number of surveys (n = 137)

Variable name	Not studied	Positive	Negative	No trend
Age	98	2	15	22
Gender (respect to male)	103	4	13	17
Education	97	25	1	14
Being a hunter	117	3	14	3
Experienced loss	116	0	16	5
Perceived risk	131	0	6	0
Livestock owner	120	4	8	5
Fear	127	0	9	1
Wolf area	129	0	6	2
Rural versus urban	132	0	3	2
Population size	129	4	1	3
Income	130	2	2	3
Knowledge	127	5	1	4

In addition to the variables in Table 2, variable values were included in ten surveys to test for an influence on attitudes towards wolves. Some surveys used a value-measurement that were wolf-specific, and assessed the relationship between this existence value of wolves and attitude (e.g., Bishop et al. 2020), while others included a more general wildlife value orientation measurement (e.g., Büssing et al. 2018; Gosling et al. 2019; Landon et al. 2020) using established wildlife value scales (e.g., Fulton et al. 1996; Manfredo et al. 2009). Several surveys found that mutualism was positively correlated with attitudes toward wolves, while domination was negatively correlated (e.g., Hermann et al. 2013; Landon et al. 2020).

### Models examining wolf presence status and respondent groups

**Fig. 4:**
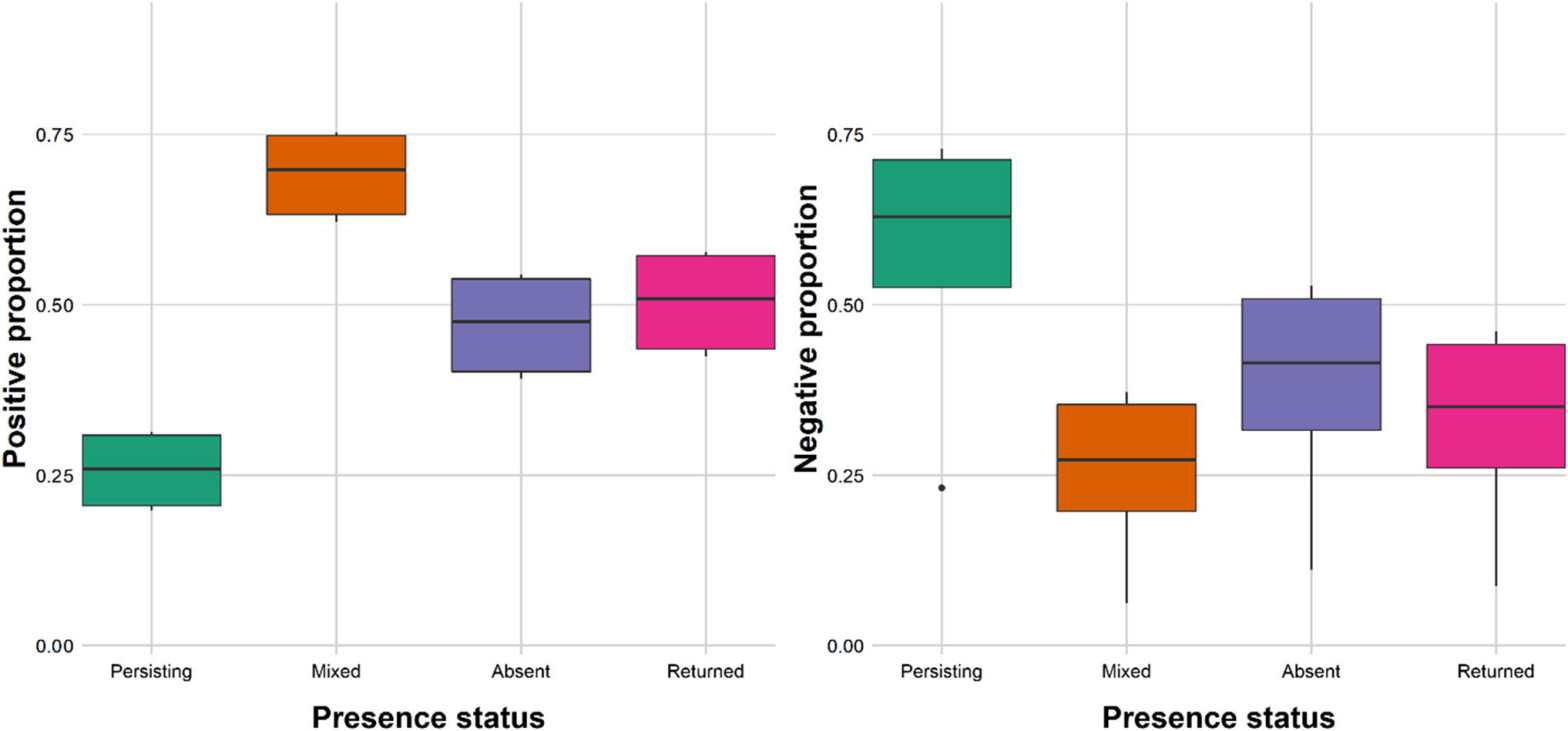
Predicted proportions from models with presence status as the predictor variable and attitude towards wolves as the response variable. In the positive proportion model, attitude is given as the proportion of the respondents that were positive towards wolves. In the negative proportion model, attitude is given as the proportion of the respondents that were negative towards wolves. The proportion is given between 0 and 1 for each presence status. On the x-axis, presence status is given, where Persisting = wolves have always been present in study area, Mixed = more than one of the presence statuses applies, Absent = wolves are absent from the study area, Returned = wolves have returned to the study area after being previously extirpated

The models showed that wolf presence influenced attitudes to some degree (Appendix C). The presence status “Persisting” was associated with more negative attitudes in both the positive and negative proportion models (Fig. 4). For the model using the positive proportion as the response variable, the mixed wolf presence status was associated with more positive attitudes than the other statuses. We observed no differences between the “Absent” and “Returned” statuses in any of the proportional models.

For the mean data model, no significant effect of presence status on attitudes was observed (Figure S1 in Appendix F).

**Fig. 5:**
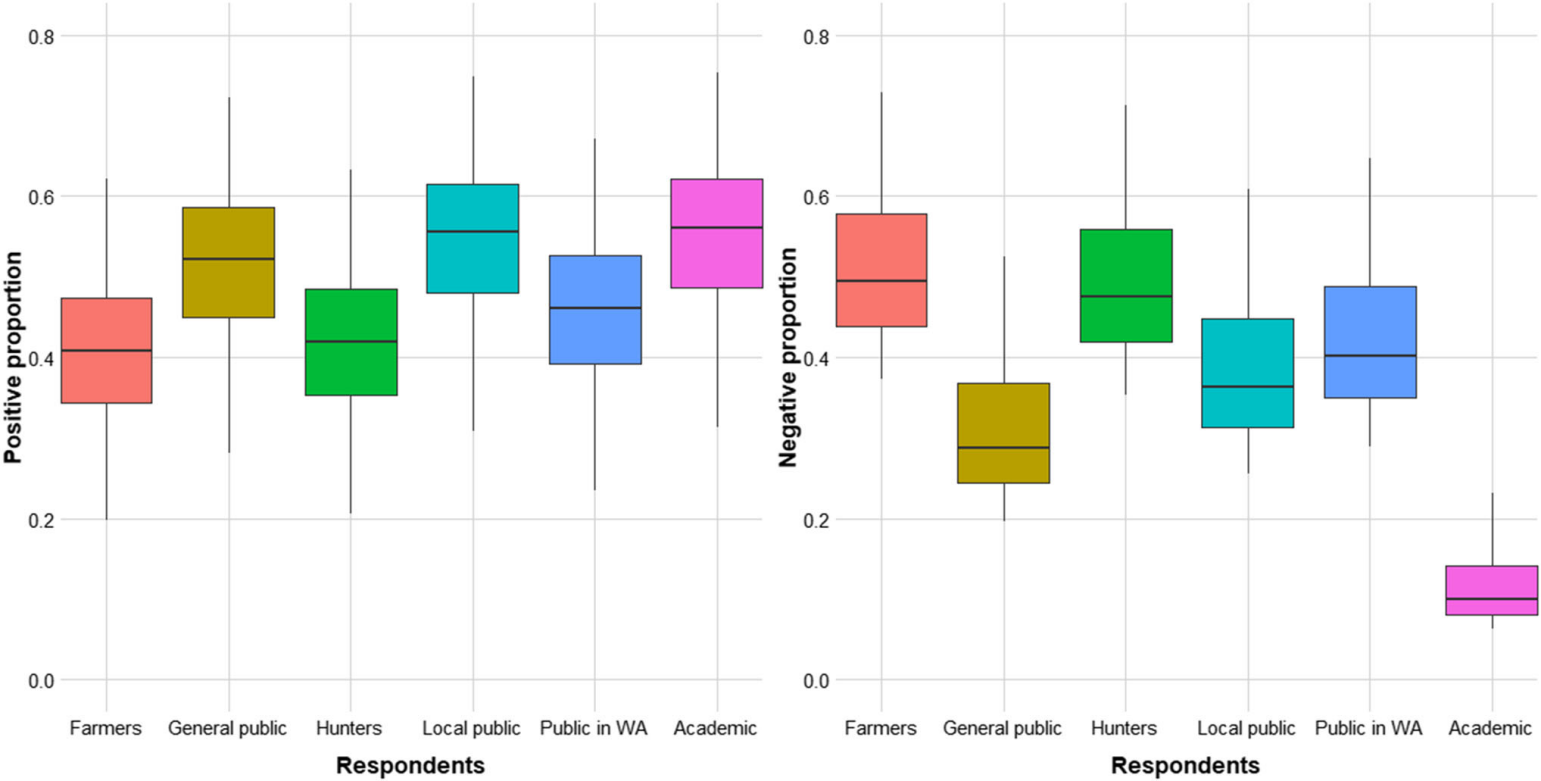
Predicted proportions from models with respondent group as the predictor variable and attitude towards wolves as the response variable. In the positive proportion model, attitude is given as the proportion of the respondents that were positive towards wolves. In the negative proportion model, attitude is given as the proportion of the respondents that were negative towards wolves. The proportion is given between 0 and 1 for each respondent group. On the x-axis, respondent groups are given, where *Public WA* public in wolf area

Academics were found to have a lower proportion of reporting negative attitudes towards wolves compared to other respondent groups (Fig. 5). As academics do not stand out in the positive proportion, this suggests that many of them must have had neutral attitudes.

**Fig. 6:**
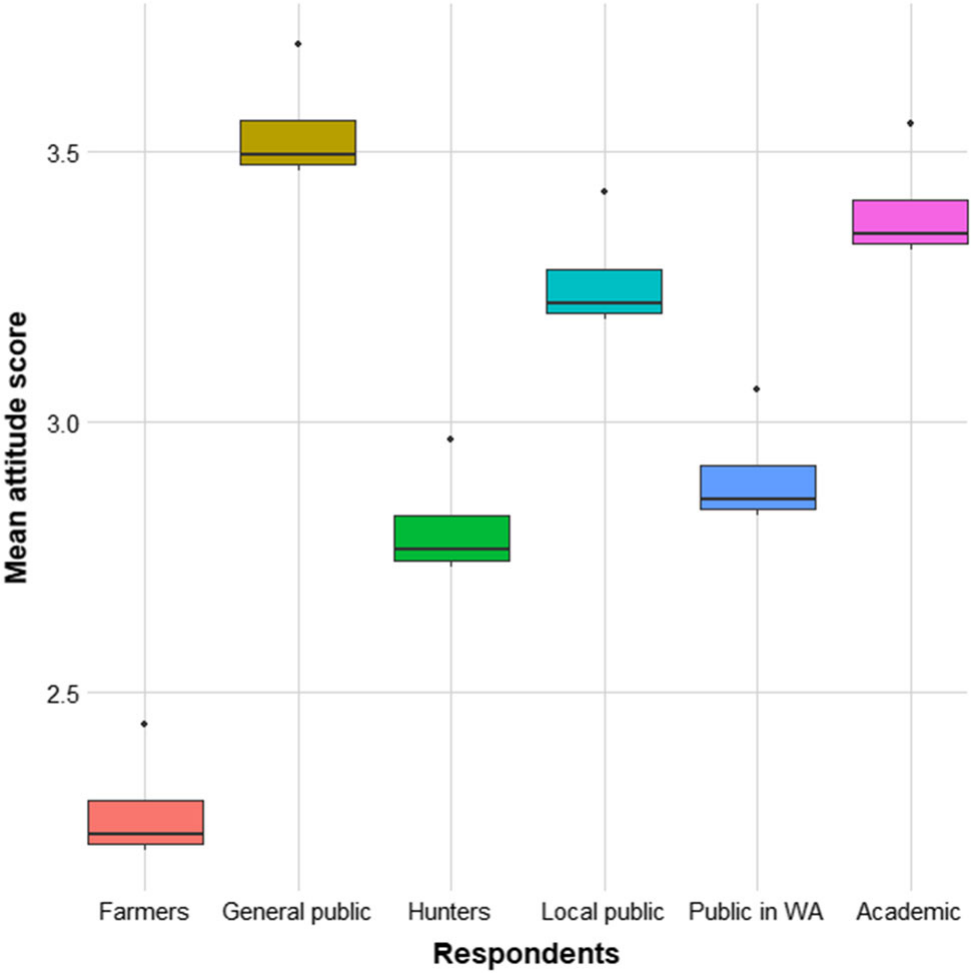
Predicted attitude towards wolves from models with respondent group as the predictor variable and attitude towards wolves as the response variable. Attitude is given as a mean score between 1 (most negative) and 5 (most positive). On the x-axis, respondent groups are given, where *Public WA* public in wolf area

For the mean data, farmers were found to be associated with most negative attitudes compared to the other respondent groups, followed by hunters and the public in wolf areas (Fig. 6). By contrast, general public and academics were found to hold the most positive attitudes.

## Discussion

### Attitudes related to presence or absence of wolves

We compared 137 different surveys to examine how attitudes towards wolves were associated with wolf presence status, and how attitudes differed among different types of respondents. Surveys reported attitudes in two different ways, and we observed slightly different results for proportional and mean data. For proportional data, people living in areas where wolves had been continuously present held more negative attitudes compared to areas where wolves had returned. This was in accordance with the findings of the systematic reviews carried out by Dressel et al. (2015) and Williams et al. (2002), who observed that time living with wolves was correlated with negative attitudes. Dressel et al. (2015), explain this as there is easy to support a hypothetical return of the wolves, but as the wolves establish, people experience an increased conflict with encounters, media attention, loss of sheep and other negative consequences (Dressel et al. 2015). However, in our study, we did not observe significant differences in attitudes between people living in areas where wolves were absent and where they had returned. This may be caused by low wolf population sizes in the re-establishment period, and/or that it could have been a short time period between their return and the time of the survey. In the “return period”, there could also be a low frequency of personal encounters (e.g., Anthony and Tarr 2019) and consequently a minimal influence of wolf recovery on public attitudes towards wolves.

### Time of exposure

Very few studies included information on when wolves had returned. Also knowledge of the accuracy of presence/absence of wolves in study areas was limited. In studies where respondents self-report whether or not they live in an area where wolves had returned, different generations may differ in their perceptions. In cases where the wolf returned e.g., 30 years ago, parents and grandparents would remember the time without wolves, in addition to an experience from when the wolf returned. Younger people (in the same area) have only experienced persisting wolves. In their systematic reviews, Williams et al. (2002) and Dressel et al. (2015) found indications of attitudes becoming more negative over time. However, the opposite effect has also been observed (Zimmermann et al. 2001; George et al. 2016; Kutal et al. 2018). To test for actual change in attitudes over time, longitudinal surveys are needed. We believe that changes in attitudes should be studied over time in the same area, preferably with the same respondents if the actual change in individuals’ attitudes are of interest, yet re-sampling the same respondents comes with challenges. Several studies have assessed change in attitudes in the same area by taking comparable samples some years apart. Bruskotter et al. (2007) looked at changes in attitudes towards wolves in Utah, and found relatively stable attitudes for all respondent groups over a 10 year period. Treves et al. (2013) observed an increase in fear of wolves and an inclination to poach wolves in their longitudinal study in Wisconsin. In Sweden, scientists observed a lower degree of support for policy goals of wolves over a 5-year period with a continued increase in wolf populations and suggested this could be linked to an increase in direct experience of the animal (Ericsson 2015). In Croatia, Majić and Bath (2010) observed that attitudes shifted towards a more neutral position, as there was both a decreased support for wolf conservation and a decrease in support to control wolf numbers.

We could also question why we do not see a pattern of people becoming used to living close to wolves over time, and thereby become more positive the longer they have coexisted. As far as we have found, very few studies actually support such a “happy coexistence”. There was however, one study in Norway in 2001 (Zimmerman et al. 2001) showing attitudes becoming more positive after the wolves had established, additional to reveal a decreased level of fear (Zimmerman et al. 2001). This study was conducted at the same time as the wolf began to recover after years of being almost extinct (Zimmerman et al. 2021), and it would be very interesting to repeat the study to see how this have developed after a longer time with wolf being present. This lack of long-term monitoring of attitudes points to the challenge that most studies just map attitudes in a snapshot, but do not investigate how or why attitudes may change over time. Studies should for instance be repeated regularly *before, at the time*, and *after* wolves’ establishment as the wolf situation changes. In Norway and in many Scandinavian and European countries, studies conducted in early 2000, represented a wolf situation where the wolf was about to increase from being extinct. Today, young people have actually been living with wolves their entire life and may hold different attitudes.

### Attitudes related to social identity

Our findings of differences in the way surveys were conducted, meant that there were unequal proportions of the different respondent groups in surveys with differing wolf presence statuses. Farmers were by far the most frequently included respondent group in surveys where wolves had been continuously present but were rarely included in surveys with other wolf presence statuses, such as returned. The model based on mean data indicated that farmers and hunters held more negative attitudes towards wolves, followed by the public in wolf areas. This was in line with what Dressel et al. (2015) found in their systematic review. Social identity can influence attitudes towards large carnivores even more strongly than personal experience or regional differences (Kellert et al. 1996; Naughton-Treves et al. 2003; Chavez et al. 2005; Lute et al. 2014).

Findings from Scandinavia, show that wolf conflicts are not necessarily driven by the presence of the wolf itself, the presence of wolf zones, nor the loss of sheep depredated by wolves (Gangaas et al. 2013, 2015). Rather, the conflict associated with large carnivores such as the wolf may as well be linked to rural cultural values such as sheep farming and strong traditions of big game hunting (Gangaas et al. 2013; Larsson et al 2022). Therefore, it is interesting that our results from this study also revealed that variables such as being a hunter, perceived risk, being a livestock owner, and experiencing fear were often associated with negative attitudes.

We found that surveys where wolves had returned, were mainly aimed at the general public who often perceived fewer negative consequences of wolves as their livelihoods were not affected in the same way. We would recommend that groups like farmers and hunter, which are the groups mostly directly affected by the presence of wolves, are included in future attitudinal studies no matter if the attitudes mapped deals with wolf being present, absent, or persistent. This would generate knowledge of attitudes among these groups also in the period before being directly affected by the presence of wolves. It could help us understand how attitudes may differ between the stages of absence to presence of the wolf.

### The impact of emotions such as fear

Fear of wolves was found to be associated with negative attitudes in all but one study (e.g., Roskaft et al. 2007; Behr et al. 2017). Fear is related to psychology, defined not only as an emotion, but also as a perception and an attitude by itself (Johansson et al. 2016). There are many different types of fear, and some of the most frequent types is fear towards animals (Broeren et al. 2011). Fear of carnivores can be caused by different emotions such as fear of entering the forest alone, to let children walk alone to the school bus in areas where carnivores occur, or the experience of losing hunting dogs to the wolf (Bjerke et al. 2003; Linnell et al. 2005). In rural areas, fear of encountering large carnivores may in certain situations prevent people from outdoor recreational activities, such as hiking (especially with dogs), berry picking or hunting (Røskaft et al. 2003; Skogen and Krange 2003). Even studies where respondents in general may be positive to conservation of large carnivores, shows that the majority do not necessarily accept large carnivores in their neighboring area (Krange et al. 2012). This phenomenon called NIMBY (Not-In-My-Back-Yard) is also known from studies related to establishment of e.g., prisons, landfills or power plants (Wexler 1996; Krange et al. 2012). Hence, emotions such as fear, can negatively affect people even if they have no experience of encounters with the feared object, here the wolf.

A previous study suggested that people were on average 1000 times more likely to accept risk which they undertook voluntarily compared to risks imposed externally (Starr 1969). Perceived risks, such as competition for big game or risk of livestock losses, were frequently included in the measured attitudes reported in the surveys (e.g., Schroeder et al. 2018; Anthony and Tarr 2019; Grima et al. 2020). Research suggests that risk and fear may reduce acceptance of management actions (Johansson et al. 2012; Slagle et al. 2012), and therefore be a driver of human intolerance towards wolves or wildlife in general (Dickman 2010; Bhatia et al. 2020).

### Negative experiences matter

Owning livestock, being a farmer, experiencing fear, or losses to predation and perceived risk were, with very few exceptions, only applicable in areas with wolves (i.e. presence status “Persisting” or “Returned”). As these variables were not included in surveys of respondents living in areas without wolves, they could not be interpreted independently of wolf presence. Nonetheless, while all three attitudinal outcomes (‘negative’, ‘positive’ and ‘neutral’) were recorded for most variables, including experiencing loss, most significant relationships reflected the negative experiences of people in wolf areas, associated with being a hunter, owning livestock and feeling fear. This emphasizes that the perceived negative consequences of living with wolves often influence attitudes negatively, as found in many surveys on large carnivores in general, and wolves in particular (e.g., Naughton-Treves et al. 2003; Karlsson and Sjöström 2007; Eriksson et al. 2015).

### Demografic variables such as age, gender and education

Age, gender and education are traditionally included in most surveys, as these variables most often have been thought to impact on people’s attitudes (Andersone and Ozoliņš 2004; Gangaas et al. 2013). In general, women are more afraid of large carnivores compared to what men report, and younger people are more positive towards having carnivores compared to elderly people. Age, gender and education are also those variables most frequently tested for an influence on attitudes towards wolves (as in attitudinal studies in general), but also those most frequently showing non-significant effects. This may be because other variables give a stronger impact when it comes to wolves. For example, Stronen et al. (2007) found that attitudes were strongly influenced by family and community. However, their study exclusively included rural-living farmers on farms which their family had owned for generations. Consequently, they argue that the influence of age and education were of less importance than social identity. Cultural differences may have a significant impact as well. In some cultures, women are not allowed or expected to be included in such surveys, and this may cause a lack of knowledge in how gender impact on attitudes in some areas. In our review, age was less likely to influence attitudes in smaller than larger geographic scale surveys. Such patterns were not found for gender or education. This might be linked to the choice of sample method, with purposive sampling being more commonly used in small-scale surveys. Another explanation could be that the way the variable is constructed may cause variation in the results. Age was given both as a categorical and continuous variable, and many studies which used the latter found no significant effect (e.g. Stronen et al. 2007; Li et al. 2015; Berry et al. 2016; Filter et al. 2020; Torres et al. 2020). The assumption of a linear relationship between age and attitudes may not suffice in all situations. Education levels differ heavily between different cultures, so this needs to be considered when comparing results in such a review. For example, while scales in some areas ranged from high school educated to PhD-level, elsewhere education may be defined in terms of being able to read and write or not (literacy, e.g., Chetri et al. 2020). There may also be a bias in some countries regarding who is able to contribute to such surveys, as analphabetism is still a challenge in some parts of the world and may prevent participation.

### Other variables that might impact attitudes

Relative to the total number of surveys, the proportion of surveys that tested variables’ influence on attitude were relatively low, as multiple surveys measured attitudes towards wolves with other objectives than to test for the influence of a range of different variables on attitude. Some surveys used structural equation models to test for the correlation between different concepts, such as risk- and benefit-based beliefs (Schroeder et al. 2018), behavioral support (Bishop et al. 2020), willingness to pay (Bishop et al. 2020), acceptability of lethal control (Straka et al. 2020), and risk perception (Landon et al. 2020). In such studies, other variables that might influence attitudes were not always included as they were not considered of interest, explaining the relative high proportion of surveys that did not report variables testing for trends summarized in Table 2. Accordingly, there might be multiple variables that either falsely identified a strong relationship between measured variables or masked the true relationship. For example, Bhatia et al. (2017) observed that religion influenced attitudes towards snow leopards when no other factors were considered, however, when accounting for variance explained by other variables the impact of religion was non-significant. Anthony and Tarr (2019) speculated that the non-significant effect of age and education in their survey might be a result of other variables more able to explain the variation.

### Wolves versus other carnivores

For all studies that included attitude measurements for species other than wolves, such as snow leopards (*Panthera uncia*), brown bears (*Ursus arctos*), hyena (*Hyaena hyaena*), wolverine (*Gulo gulo*), or Eurasian lynx (*Lynx lynx*) (e.g., Behmanesh et al. 2019; Trajce et al. 2019; Augugliaro et al. 2020), attitudes were exclusively more positive than attitudes towards wolves. Multiple surveys in Asia included measurements of respondents’ attitudes towards snow leopards in addition to wolves and reported significantly more positive attitudes towards snow leopards than wolves (respondents included in these surveys were only members of public in wolf areas or farmers). Another interesting finding, was how management actions had an impact on the attitudes towards wolves, as a study of Samelius et al. (2020) revealed more negative attitudes towards wolves after installing fences against wolves and snow leopards, despite reduced livestock predation (Samelius et al. 2020). Similarly, Suryawanshi et al. (2013) reported that local people perceived wolves to have a greater negative impact on livestock than snow leopards, even though the data on actual damage suggested otherwise.

The difference in reported attitudes towards wolves compared to attitudes towards other carnivores, is a continuous discussion. The phenomenon is being explained by a variety of theories from hypothesis that the old stories and myths of the “big bad wolf” is causing a hatred against the wolf, to challenges among farmers and hunters, to the life history of the wolves. As far as we know, there has been no studies looking at whether or not the wolves’ life history may impact on peoples’ thoughts of having wolves in their home areas, but wolves may be experienced as more exposed to people as they live in family groups and are active year around. People may observe tracks on the snow, carcasses of large prey, and wolves can be seen as frightening when they come chasing as a pack, or killing hunting dogs close to people’s homes. Bears, who kills more people during a year than wolves do, are sleeping the whole winter, and rarely leave tracks on the snow. They occur solitarily and are not chasing dogs or encountering people in the same way as wolves may. This is just personal questions and speculations from our side, so there may be other explanations, but there is historically a much stronger aversion to wolves compared to other carnivore species which deserves to be better explained.

### The challenge of measuring attitudes, and the expectation of a related behavior

Some surveys reported neutral attitudes. Psychologists have defined attitude as the evaluation of an object with a degree of favor or disfavor (Eagly and Chaiken 1993), i.e. it has a direction. The reviewed surveys included a range of measurements, and, in some, attitudes, tolerance or acceptance were measured in a way that allowed for neutral answers. The concepts of tolerance and acceptance have been discussed in the literature (e.g., Bruskotter and Fulton 2012; Treves 2012; Bruskotter and Wilson 2014). Tolerance may be viewed as the passive acceptance of wildlife populations (Treves and Bruskotter 2014), and therefore an observed neutral attitude may be interpreted as tolerance. But as attitude and behavior are not synonymous (Eagly and Chaiken 1993; Fishbein and Ajzen 2010; Heberlein 2012), tolerant attitudes do not necessarily lead to tolerant behavior. Many of the more recent surveys that we reviewed tested for a relationship between attitude and behavioral intention (e.g., Bruskotter et al. 2015; Bhatia et al. 2020; Bishop et al. 2020; Niemiec et al. 2020), as well as past behavior (e.g., Bhatia et al. 2020). Some used two individual measures for acceptance and attitude (e.g., Bruskotter et al. 2015). While general attitudes towards wolves and attitudes towards their reintroduction or the management of an existing population likely correlate (e.g., Bath 1989; Enck and Brown 2002), exceptions do also exist. For example, Sakurai et al. (2020) found no significant association between attitude towards wolves and support for their reintroduction, and Grima et al. (2020) observed respondents to have more positive attitudes towards wolves than towards wolf reintroduction. The composition of the measurements also varies when it comes to items included in relation to cognitive and affective components. Some included terms for normative beliefs and beliefs about wolves in the measure of attitude, while others have argued that beliefs and attitudes should be measured separately (Glikman et al. 2011; Verplanken et al. 1998).

### Challenges between proportional data and mean data

Discrepancies between proportional and mean data might arise for a number of reasons such as differences in sampling methods and sample sizes between surveys. They may also be a consequence of the two different measurement scales. The mean data measured attitudes over a wider scale, allowing for more variance in the data and thus perhaps a more nuanced overall picture of effects compared to the usually more binary measured effect of e.g., positive/negative attitudes in the proportional data. Varying study designs, including ways of measuring attitude, may cause decrements in results. For example, the lack of a standardized system to measure conflict intensity suppressed spatio-temporal patterns in a survey aiming to examine conflict escalation over time (Anand and Radhakrishna 2017). In addition, the different use of terminology in questions may give contrary conclusions (Lewis et al. 2012; Berry et al. 2016), while the use of different sampling methods (i.e. random, purposive, and convenient samples) complicates interpretation. Nonetheless, a systematic review of attitudes can give an overview of differences in attitudes and how they are surveyed. This may reveal limitations on how comparable measurements are and provide suggestions for future improvements. A systematic review is less suitable for guiding management decisions, as country-specific considerations are needed in order to apply appropriate conflict-mitigation tools, as demonstrated by Trajce et al. (2019). Even at local scales, people may hold different attitudes (Glikman et al. 2010; Sponarski et al. 2013; Stauder et al. 2020), and thus spatial heterogeneity needs to be considered in attitude surveys to facilitate situation-specific strategies.

## Recommendations for future studies

This study provides an overview of research exploring attitudes towards wolves, of which we conclude that there are challenges related to making comparisons or show trends, due to the wide range of sampling methods, variables tested and ways of measuring and reporting attitudes. For future research, we recommend a standardized system for conducting attitude surveys of wildlife species to facilitate spatial–temporal analysis of perceptions. By using standardized variables, there will be possible to compare studies independent of where they are conducted, and there would be a huge contribution if studies were done before, under and after wolf recoveries (Table 3). Social science competence is needed in order to get more comparable measurements, ideally including both cognition and emotions to improve predictions of peoples’ responses to e.g., wolf management actions (Schroeder et al 2018; Jacobs and Vaske 2019; Bishop et al. 2020; Straka et al. 2020; Dheer et al. 2021). We suggest to including qualitative studies, by doing e.g. in-depth-interviews of key persons, or arranging workshops to get a broader understanding of peoples thoughts, feelings and get into the complexity of the conflicts.

**Table 3:** A suggestion of the variables (listed under Variable name) that should be included in the future studies, based on those variables we found to be most associated with attitudes towards wolf. Studies should also be conducted in time perspective before, under and after wolves have established (suggested on the top row of the table)

Variable name	Wolves being persistent	Wolves recovering after extinction	Wolves absent
Gender	X	X	X
Education	Needs to be adapted to the context of the study, and national education systems		
Being a hunter	X	X	X
Experienced loss	X	X	
Livestock owner	X	X	X
Fear	X	X	X
Living within wolf area	X	X	

We found that groups directly affected by wolves like hunters and farmers, and the respondents' feeling of fear (e.g., Johansson et al. 2019) was important as explanatory variables of attitudes, additional to demographic variables such as education level. We also recommend including questions related to value orientations (e.g., being ecocentric or anthropocentric) to better understand the basic values that attitudes are based on Jürgens et al. (2023) and Barmoen et al. (2022). Last, to make it feasible to compare measured attitudes across studies, attitude measurements need to be reported in a standardized way, either as mean data or proportional data. If proportional data are chosen, either all categories need to be reported (positive, negative and neutral) or all surveys need to agree upon which of them to use.

## Recommendations for future conservation

As there already are numerous studies which has aimed at mapping attitudes towards wolves in general, we now suggest emphasizing future research on understanding the *reason why* we observe the attitudes that we do, *do attitudes change over time*, and *what is needed to increase the chance of coexistence between the wolves and humans* (Pettersson et al 2022). Also in future surveys, we will recommend including those groups of people who are directly affected by having wolves (e.g., hunters and farmers), either by doing geospatial surveys to ensure including areas where wolves actually live (Pettersson et al 2022) or include the NIMBY effect (von Essen and Allen 2020). Hunters and farmers have shown to be the ones who can impact on wolf management or wolf conservation as they often represent strong voices in local societies (Treves and Martin 2011; Larsson et al 2022). We will also propose that future research focus on understanding the attitudinal barriers and find opportunities of achieving a wolf-human coexistence by examining attitudes of different management actions that can be supported, and also to a higher extent by using qualitative studies (Jacobs et al. 2014; Din et al 2017; Johansson et al 2019; Larsson et al 2022; Pettersson et al. 2022).

In areas where wolves are to be reestablished, or natural reestablishment are expected, we recommend management authorities and politicians to develop a communication- and action plan to better prepare the local human population for which consequences presence of wolves may cause. In wolf areas, we will emphasize the importance of close dialogue on how to deal with wolves in the local area.

## Supplementary Information

Below is the link to the electronic supplementary material.

Supplementary file1 (PDF 675 KB)
