# Supplementary material for: Living with wolves: A worldwide systematic review of attitudes
Source: Ambio. 2024 Jun 4;53(10):1414–32. doi: 10.1007/s13280-024-02036-1 (PMC11383909; doi:10.1007/s13280-024-02036-1)
Supplement: Supplementary file 1 — Supplementary file1 (PDF 675 KB) [file 13280_2024_2036_MOESM1_ESM.pdf]

Ambio - Supplementary Information

This supplementary information has not been peer reviewed.

Title: Living with wolves - A worldwide systematic review of attitudes

Authors: Magnus Barmoen, Kim Magnus Bærum, Kristin E. Mathiesen

Supporting material

Appendix A: All included studies

Survey year	Country	Citation
2017	Mongolia	(Augugliaro et al., 2020)
2017	India	(Bhatia et al., 2020)
2014	Canada	(Bishop et al., 2020)
2019	USA	(Casola et al., 2020)
2014	Nepal	(Chetri et al., 2020)
2019	Germany	(Filter et al., 2020)
2017	USA	(Grima et al., 2020)
2018	Mixed	(Grossmann et al., 2020)
2017	Russia	(Kirilyuk & Ke, 2020)
2016	Nepal	(Kusi et al., 2020)
2016	USA	(Landon et al., 2020)
2019	USA	(Niemiec et al., 2020)
2019	Japan	(Sakurai et al., 2020)
2014	Mongolia	(Samelius et al., 2020)
2018	Italy	(Stauder et al., 2020)
2019	Portugal	(Torres et al., 2020)
2017	Hungary	(Anthony & Tarr, 2019)
2018	Germany	(Arbieu et al., 2019)
2017	Iran	(Behmanesh et al., 2019)
2018	Germany	(Bussing et al., 2019)
2017	Poland	(Gosling et al., 2019)
2016	Pakistan	(Khan et al., 2019)
2009	Albania and Macedonia	(Trajce et al., 2019)
2014	USA	(Bruskotter et al., 2018)
2016	Slovenia	(Oražem & Tomažič, 2018)
2013	USA	(Schroeder et al., 2018)
2015	Switzerland	(Behr et al., 2017)
2014	India	(Bhatia et al., 2017)
2012	Afghanistan and Pakistan and Tajikistan	(Din et al., 2017)
2014	Iran	(Farhadinia et al., 2017)
2014	Denmark	(Højberg et al., 2017)
2010	Norway	(Krange et al., 2017)
2014	Pakistan	(Ahmad et al., 2016)
2013	USA	(Berry et al., 2016)
2014	USA	(George et al., 2016)
2015	Norway	(Kaltenborn & Brainerd, 2016)
2013	China	(Alexander et al., 2015)
2010	Sweden	(Berg & Solevid, 2015)
2011	USA	(Browne-Nunez et al., 2015)

2011	USA	(Bruskotter et al., 2015)
2004	Sweden	(Eriksson et al., 2015)
2014	Sweden	(Gangaas et al., 2015)
2011	China	(Li et al., 2015)
2013	China	(Shi et al., 2015)
2012	China	(Xu et al., 2015)
2013	Netherlands	(Jacobs et al., 2014)
2013	USA	(Lute et al., 2014)
2013	USA	(Smith et al., 2014)
2009	Canada	(Sponarski et al., 2014)
2013	India	(Suryawanshi et al., 2014)
2012	Pakistan	(Din et al., 2013)
2011	Germany	(Hermann & Menzel, 2013)
2011	Germany	(Hermann et al., 2013)
2012	Norway	(Kaltenborn et al., 2013)
2007	Italy	(Glikman et al., 2011)
2007	Greece and Italy	(Hovardas & Korfiatis, 2012)
2010	Portugal	(Milheiras & Hodge, 2011)
2009	USA	(Shelley et al., 2011)
2004	USA	(Treves & Martin, 2011)
2009	India	(Agarwala et al., 2010)
2009	North Macedonia	(Lescureux & Linnell, 2010)
1999	Croatia	(Majić & Bath, 2010)
2009	Slovakia	(Prokop & Tunnicliffe, 2010)
2008	USA	(Wilson & Bruskotter, 2009)
1994	USA	(Bruskotter et al., 2007)
2006	Sweden	(Karlsson & Sjostrom, 2007)
2005	Scotland	(Nilsen et al., 2007)
2000	Norway	(Roskaft et al., 2007)
2002	Canada	(Stronen et al., 2007)
2004	Sweden	(Ednarsson, 2006)
1999	USA	(Chavez et al., 2005)
2003	Latvia	(Andersone & Ozoliņš, 2004)
2000	Norway	(Kleiven et al., 2004)
2001	Sweden	(Ericsson & Heberlein, 2003)
2001	USA	(Naughton-Treves et al., 2003)
2001	Mexico	(Rodriguez et al., 2003)
1999	USA	(Enck & Brown, 2002)
1998	Norway	(Kaltenborn et al., 1999)
1997	Norway	(Tore Bjerke et al., 1998)
1997	Norway	(T. Bjerke et al., 1998)
1996	USA	(Schoenecker & Shaw, 1997)
1995	Canada	(Lohr et al., 1996)
1994	USA	(Pate et al., 1996)
1991	USA	(Kellert et al., 1996)
1987	USA	(Bath, 1989)
1988	USA	(Tucker & Pletscher, 1989)
1978	USA	(Kellert, 1985)

Appendix B: Categorization criteria for respondent groups. Based on Dressel et al. (2015), modified to fit data.

Sample group	Definition
General public	Respondents of large regions (states) and national samples
Local public	Samples of limited geographical area (counties, districts), groups sampled as control group outside wolf territory
Urban public	Residents of cities and urban areas
Public in wolf area	Residents of an area where wolves permanently present and which were therefore selected for the study
Farmers	Including shepherds, livestock holders, cereal farmers
Academics	University students, school pupils, ecology researchers, managers responsible for wolf management selected for the study because of that
Hunters	Hunters, gamekeepers

Appendix C: The table shows parameter estimates with standard error, z value and p value as obtained from the model used to explore the influence of "respondent group" and "presence status" on proportional positive attitude

Variable	Estimate	Std. Error	z value	p value
Intercept	-1.39892	0.23695	-5.904	>0.001
Presence status mixed	1.89648	0.46321	4.094	>0.001
Presence status Absent	0.95880	0.49107	1.952	0.051
Presence status Returned	1.09439	0.30142	3.631	>0.001
Respondents General public	0.46364	0.36059	1.286	0.199
Respondents Hunters	0.04389	0.40557	0.108	0.914
Respondents Local public	0.59359	0.51944	1.143	0.253
Respondents Public in wolf area	0.21906	0.32730	0.669	0.503
Respondents Academics	0.61707	0.58496	1.055	0.291

Appendix D: The table shows parameter estimates with standard error, z value and p-value as obtained from the model used to explore the influence of "respondent group" and "presence status" on proportional negative attitude

Variable	Estimate	Std. Error	z value	p value
Intercept	0.98858	0.19718	5.014	>0.001
Presence status mixed	-1.50978	0.41897	-3.604	>0.001
Presence status Absent	-0.87382	0.43692	-2.000	0.046
Presence status Returned	-1.14430	0.25696	-4.453	>0.001
Respondents General public	-0.88657	0.31832	-2.785	0.005
Respondents Hunters	-0.07986	0.36961	-0.216	0.829
Respondents Local public	-0.54554	0.46077	-1.184	0.236
Respondents Public in wolf area	-0.37808	0.27244	-1.388	0.165
Respondents Academics	-2.18952	0.54239	-4.037	>0.001

Appendix E: The table shows parameter estimates with standard error, t value as obtained from the model used to explore the influence of "respondent group" and "presence status" on mean attitude scores

Variable	Value	Std. Error	t value
Intercept	2.4406	0.2207	11.056

Presence status mixed	-0.1889	0.5387	-0.351
Presence status Absent	-0.2351	0.2292	-1.026
Presence status Returned	-0.2193	0.1936	-1.132
Respondents General public	1.2583	0.2637	4.773
Respondents Hunters	0.5267	0.2961	1.778
Respondents Local public	0.9839	0.2939	3.348
Respondents Public in wolf area	0.6209	0.2934	2.1160
Respondents Academics	1.1124	0.2866	3.881

Appendix F:

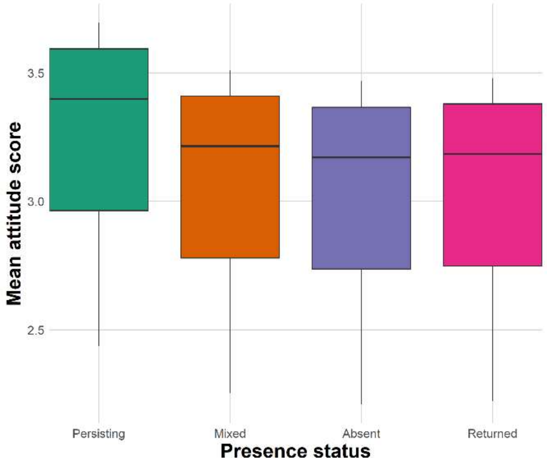

Figure S1: Predicted attitude towards wolves from models with presence status as the predictor variable and attitude towards wolves as the response variable. Attitude is given as a mean score between 1 (most negative) and 5 (most positive). On the x-axis, presence status is given, where Persisting = wolves have always been present in study area, Mixed = more than one of the presence statuses applies, Absent = wolves are absent from the study area, Returned = wolves have returned to the study area having been previously extirpated.
